# Phosphoserine-86-HSPB1 (pS86-HSPB1) is cytoplasmic and highly induced in rat myometrium at labour

**DOI:** 10.1007/s00418-022-02158-1

**Published:** 2022-10-19

**Authors:** E. I. Miskiewicz, A. Olaloku, B. K. MacPhee, D. J. MacPhee

**Affiliations:** 1 Department of Veterinary Biomedical Sciences, Western College of Veterinary Medicine, University of Saskatchewan, 52 Campus Dr, Saskatoon, SK S7N 5B4 Canada; 2 One Reproductive Health Research Group, University of Saskatchewan, Saskatoon, SK S7N 5B4 Canada

**Keywords:** HSPB1, Myometrium, Labour, Actin, VASP, Adhesion, Cytoskeleton

## Abstract

Uterine myocytes during pregnancy proceed through a series of adaptations and collectively transform into a powerfully contractile tissue by term. Previous work has indicated that members of the heat shock protein (HSP) B family of stress proteins are associated with the process of adaptation and transformation. Utilizing immunoblot analyses, widefield epifluorescence and total internal reflection (TIRF) microscopy, this study investigated the temporal and spatial detection of HSPB1 phosphorylated on serine-86 (pS86-HSPB1) in rat myometrium during pregnancy, the role of uterine distension in regulation of pS86-HSPB1, and the comparative localization with pS15-HSPB1 in rat myometrial tissue as well as in an immortalized human myometrial cell line. Immunoblot detection of pS86-HSPB1 was significantly elevated during late pregnancy and labour. In particular, pS86-HSPB1 was significantly increased at day (d)22 and d23 (labour) compared with all other timepoints assessed. Localization of pS86-HSPB1 in myometrium became prominent at d22 and d23 with cytoplasmic detection around myometrial cell nuclei. Furthermore, pS86-HSPB1 detection was found to be significantly elevated in the gravid rat uterine myometrium compared with the non-gravid tissue at d19 and d23. Both widefield epifluorescence and TIRF microscopy examination of human myometrial cells demonstrated that pS15-HSPB1 was prominently localized to focal adhesions, while pS82-HSPB1 (homologous to rodent pS86-HSPB1) was primarily located in the cell cytoplasm. Our data demonstrate that levels of phosphorylated HSPB1 increase just prior to and during labour, and that uterine distension is a stress-inducing signal for HSPB1 phosphorylation. The exact roles of these phosphorylated forms in myometrial cells remain to be determined.

**Supplementary Information:**

The online version contains supplementary material available at 10.1007/s00418-022-02158-1.

## Introduction

Parturition requires the coordinated production and regulation of systemic and local signaling cascades from many compartments that ultimately must converge for the timely delivery of an infant at term when it is physiologically capable of extra-uterine life (Menon et al. 2016). These compartments include the foetal hypothalamic–pituitary–adrenal axis, the amnion and placenta, and the decidua and uterine smooth muscle or myometrium. The myometrium is an immune regulatory tissue that produces cytokines and chemotactic stimuli for the infiltration of leucocytes that supply signals for labour (Shynlova et al. 2020). It consists of myocytes that during pregnancy proceed through a series of changes such as increased proliferation during early gestation, hypertrophy and extracellular matrix (ECM) remodelling, induced in part by foetal growth-induced distension, and transformation into a powerfully contractile tissue by term (MacPhee and Miskiewicz 2017).

Much of what we have learned regarding myometrial differentiation and adaptation over pregnancy has originated from the use of animal models such as the rat (Shynlova et al. 2013). The rat model is advantageous for the collection of myometrial tissue at precise timepoints over the entirety of gestation and has been used to assess the impact of uterine distension on the expression of key parturition-associated genes (Shynlova et al. 2020). While differences in the processes leading to parturition in humans and rats exist, such as in progesterone withdrawal in the maternal circulation, there are also similarities, including the consistent involvement of oxytocin receptors and connexin-43 containing gap junctions in the regulation of uterine contractility (Mitchell and Taggart 2009; Bonney 2013; Shynlova et al. 2013).

The small heat shock protein B (HSPB) family is composed of ten small-molecular-weight proteins (15–43 kDa; Collier and Benesch 2020) that are key for cellular homoeostasis and are induced by physiological stressors such as temperature, oxidative stress and biophysical forces (reviewed by Mymrikov et al. 2011; Kampinga and Garrido 2012). Canonically, HSPB members act as ATP-independent molecular chaperones that bind to unfolded substrate proteins following stress and prevent them from irreversible aggregation until they are passed on to the ATP-dependent chaperone networks (e.g. HSP70/HSPA family) for refolding (Collier and Benesch 2020). However, they also interact with client proteins in physiological processes such as cell death regulation, cytoskeletal remodelling and immune system activation (Acunzo et al. 2012; van Noort et al. 2012; Wettstein et al. 2012). Importantly, many of the HSPB members can heteroligomerize with one another, adding greater functional complexity (Zantema et al. 1992; Fontaine et al. 2005).

The domain organization of the HSPB family members is well known and has been reviewed elsewhere (Haslbeck et al. 2015). Among post-translational modifications of the HSPB family, phosphorylation of these proteins on serine (Ser) residues in the NH_2_-terminal region is critical for regulation of structure and function (Mymrikov et al. 2011). In fact, it is known to be one of the earliest events induced by a stress, including heat shock, oxidative stress and stimuli such as cytokines and growth factors (Landry et al. 1992). Three serine phosphorylation sites have been recognized in human HSPB1 (Kostenko and Moens 2009): Ser-15 (pS15-HSPB1), Ser-78 (pS78-HSPB1) and Ser-82 (pS82-HSPB1). In contrast, two phosphorylation sites were reported for HSPB1 in rodents: Ser-15 and Ser-86 (pS86-HSPB1), the latter homologous to Ser-82 in humans and Ser-90 in hamsters (Gaestel et al. 1991). HSPB1 phosphorylation results in dissociation of large oligomers of HSPB1 and chaperoning activity (Mymrikov et al. 2011; Jovcevski et al. 2015; 2017). pS15-HSPB1 may directly bind with actin microfilaments (Lambert et al. 1999), and past reports have indicated a role for HSPB1 in actin polymerization, remodelling and even cross-bridge cycling in smooth muscle cells (Lavoie et al. 1993a, b; Benndorf et al. 1994; Guay et al. 1997; Ibitayo et al. 1999). During et al. (2007) demonstrated that HSPB1 is an actin monomer sequestering protein and that HSPB1 phosphorylation enhances actin filament assembly.

We previously reported that *Hspb1* mRNA expression was significantly increased during late pregnancy in the rat myometrium, that pS15-HSPB1 detection significantly increased during late pregnancy and labour, and that pS15-HSPB1 was induced by uterine distension (White et al. 2005; White and MacPhee 2011). Furthermore, pS15-HSPB1 had a predominantly membrane-associated localization in situ within cells of the circular and longitudinal muscle layers; however, pS86-HSPB1 was not examined. Thus, the objectives of this study were to investigate the temporal and spatial detection of pS86-HSPB1 in rat myometrium during pregnancy, the role of uterine distension in regulation of this phosphorylated form of HSPB1, and the comparative localization with pS15-HSPB1 in myometrial cells.

## Materials and methods

### Animals and ethics approval

Sprague Dawley rats were individually housed by the Institutional Animal Care Unit under standard environmental conditions (12 h light/12 h dark cycle). Rats had access to water ad libitum and were maintained on LabDiet Prolab (RMH 3000; PMI Nutrition International, Brentwood, MO, USA). For all experiments, virgin female rats were mated with stud males and the presence of a vaginal plug the morning after mating was designated day (d)1 of pregnancy. The time of delivery under these standard conditions was d23. All experiments adhered to institutional and national standards for the care and welfare of animals and were granted ethical approval by the institutional animal care committee under protocols 08–02-DM to 11–02-DM.

### Tissue collection

Individual female rats were euthanized utilizing carbon dioxide gas asphyxiation. Tissue samples were collected from animals at ten timepoints including non-pregnant (NP) and throughout gestation at d6, d12, d15, d17, d19, d21, d22 and d23 (labour) as well as 1 day post-partum (PP). The labour samples collected on d23 were taken only during active labour when the mother had delivered two to three pups.

For immunoblot analysis, the uterine horns were dissected in cold phosphate-buffered saline solution (PBS; pH 7.4). Myometrial tissue was then isolated as previously described (Nicoletti et al. 2016). All collected samples were flash frozen in liquid nitrogen and stored at −80 °C. For immunofluorescence detection, a portion of the rat uterine horn was fixed in 4% paraformaldehyde (PFA) in PBS overnight at room temperature. Tissues were processed, paraffin embedded, sectioned and mounted on microscope slides as previously outlined (Bhatti et al. 2019).

To assess the effect of uterine distension on HSPB1 expression, a unilaterally pregnant rat model was utilized. This model has been previously used to study the effect of uterine distension or stretch on numerous genes and proteins including the gap junction protein GJA1 (Ou et al. 1997), the heat shock protein CRYAB (Nicoletti et al. 2016) and signaling kinases such as MAPK14 (Oldenhof et al. 2002). Virgin female rats were anaesthetized and underwent a unilateral tubal ligation procedure as previously described (White and MacPhee 2011). Animals were monitored post-operatively and allowed to recover for at least 5 days before matings were attempted. The model results in pregnant rats each having a gravid (distended) and non-gravid (empty, non-distended control) uterine horn for analyses. Samples of gravid and non-gravid horns were collected on gestational d19 and d23 and tissues processed as described above.

### Cell culture

The hTERT-HM human myometrium-derived cell line was established via stable transfection of human myometrial cells with expression vectors containing the human telomerase reverse transcriptase (hTERT) for telomere length maintenance and immortalization (Condon et al. 2002). These cells retain myometrial cell characteristics such as the expression of CNN1 (calponin) and OXTR proteins (Condon et al. 2002). hTERT-HM cells were maintained in 75 cm^2^ culture flasks at 37 °C and 5% CO_2_ in air using DMEM/F12 medium with l-glutamine and 15 mM HEPES (11330–032; ThermoFisher) plus 10% foetal bovine serum (FBS; 12483–020; ThermoFisher) and 1% penicillin–streptomycin (P/S; 15140–122; ThermoFisher). The medium was refreshed every 24 h, and cells were passaged when they reached ~ 80% confluence using trypsin (0.05% v/v trypsin–EDTA, 15400–054; ThermoFisher). Cells were subsequently seeded (1 × 10^5^ cells) on to 22 mm × 22 mm sterile glass coverslips placed within six-well tissue culture plates for widefield epifluorescence or in 35 mm glass-bottom culture dishes (P35G-1.5–14-C; MatTek Corporation) for total internal reflection fluorescence microscopy (TIRF) and cultured in 2 mL of medium for 24 h prior to experiments.

### Immunoblot analysis

Tissue samples (*n* = 4 per timepoint) were homogenized using a Precellys Bead Mill in radioimmunoprecipitation assay (RIPA) lysis buffer containing protease and phosphatase inhibitors as previously described (Nicoletti et al. 2016). Sample protein concentrations were then determined using the Bio-Rad Protein Assay Kit II (500–0002; Bio-Rad). Protein samples (20 μg per lane) were electrophoretically separated on 12% SDS–polyacrylamide gels followed by electroblotting to 0.2 μm nitrocellulose membranes (162–0097; Bio-Rad). Membranes were then stained with a Reversible Protein Stain kit (24580; ThermoFisher), according to the manufacturer’s instructions, to verify protein transfer.

Nitrocellulose membranes were rinsed in Tris-buffered saline containing Tween 20 (TBST) and then incubated in a blocking solution composed of 5% non-fat dry milk (w/v) or 5% bovine serum albumin (10735078001; Roche Diagnostics) in TBST. All anti-sera used for analyses (Table 1) were diluted in blocking solution. Blots were incubated with primary anti-sera overnight at 4 °C with constant shaking, rinsed with TBST and probed with an appropriate horseradish peroxidase-conjugated secondary antibody for one hour at room temperature. Protein detection was accomplished using a SuperSignal West Pico Plus chemiluminescence substrate detection system (34580; ThermoFisher), and multiple exposures were acquired using a Bio-Rad ChemiDoc MP digital imaging system. All membranes were subsequently re-probed for glyceraldehyde 3-phosphate dehydrogenase (GAPDH) protein expression, which served as a loading control, using a rabbit polyclonal GAPDH-specific antibody (Table 1).

**Table 1:** Anti-sera utilized for immunofluorescence (IF) and immunoblot (IB) analysis

Anti-sera	Method and dilution used	Company and catalogue number
Rabbit anti-GAPDH	IB: 1:10,000	Abcam; ab9485
Rabbit anti-pSer82/86-HSPB1	IB: 1:10,000	Abcam; ab32035
Rabbit anti-pSer82/86-HSPB1	IF-T: 1:200	Sigma Aldrich; SAB4504434
Rabbit anti-pSer82/86-HSPB1	IF-C: 1:100	Cell Signaling Technology; 9709
Rabbit anti-pSer15-HSPB1	IF-T: 1:200	ThermoFisher; PA1-016
Rabbit anti-pSer15-HSPB1	IF-C: 1:100	StressMarq; SPC-1263
Mouse anti-VASP	IF-C: 1:25	BD Biosciences; 610447
Mouse anti-zyxin	IF-T: 1:25	BD Biosciences; 610521
Goat anti-rabbit HRP	IB: 1:10,000	Promega; W4011
Donkey anti-rabbit AF488	IF-T and IF-C: 1:150	Jackson ImmunoResearch; 711–545-152
Donkey anti-mouse RRX	IF-T and IF-C: 1:150	Jackson ImmunoResearch; 715–295-150
ChromPure mouse IgG	IF-T and IF-C: *	Jackson ImmunoResearch; 015–000-003
ChromPure rabbit IgG	IF-T and IF-C: *	Jackson ImmunoResearch; 011–000-003

*AF488* Alexa Fluor-488, *RRX* Rhodamine-Red-X, *HRP* horseradish peroxidase, *IF-T* immunofluorescence analysis of tissue sections, *IF-C* immunofluorescence analysis of cells

*Matched to concentration of primary anti-sera utilized

### Immunofluorescence analysis

For immunofluorescence detection in uterine tissues, tissue sections (*n* = 3 per timepoint) were deparaffinized and rehydrated and then underwent epitope retrieval as previously described (Nicoletti et al. 2016). Sections were incubated for 1 h in a blocking solution consisting of 5% normal goat serum, 1% normal horse serum, and 1% FBS in PBS. Sections were then incubated overnight at 4 °C with primary anti-serum or non-specific IgG (Table 1) used at the same concentration as primary anti-serum to serve as a negative control. Sections were washed with PBS then incubated with appropriate secondary anti-serum (Table 1) for 1 h at room temperature. Following washes in PBS containing 0.02% Tween 20, sections were either blocked again and the immunofluorescence procedure repeated with another pair of primary and secondary anti-sera (for co-localization analysis) or sections were mounted in ProLong Diamond Anti-Fade Reagent with DAPI (P36931; ThermoFisher).

For immunofluorescence detection in hTERT-HM cells, cells were fixed with 4% PFA in PBS for 5 min at room temperature and then washed with PBS. Cells were then treated with PBS containing 0.1% Triton X-100 for 15 min and subsequently with the blocking solution for 30 min at room temperature. Combinations of primary and secondary anti-sera as well as non-specific IgG (Table 1) were then used for immunofluorescence as described above. Following washes in PBS containing 0.1% Triton X-100, cells on coverslips were mounted using ProLong Diamond Anti-Fade Reagent with DAPI or cells in glass-bottom dishes were stored in PBS for TIRF image acquisition.

For all widefield epifluorescence imaging, an Olympus IX83 microscope equipped with 40× UplanSApo [numerical aperture (NA) 0.95], 60× oil UPlanSApo (NA 1.35) and 100× oil UPlanSApo (NA 1.40) objectives, and an Andor Zyla 4.2 sCMOS camera (2048 × 2048 pixel array; Andor USA), X-Cite XLED light source (Excelitas Technologies USA) and CellSens software (Olympus) were used for image acquisition. Post-image acquisition deconvolution of epifluorescence images for subsequent co-localization analysis utilized *z*-stacks of 5 × 0.25-μm-thick optical slices captured with the 100× oil objective and five repetitions per slice of constrained iterative restoration using the advanced maximum likelihood estimation algorithm. For TIRF microscopy, the IX83 system above was also equipped with a single-line motorized cellTIRF illuminator with automatic theoretical critical angle determination and setting, a multi-laser combiner (TOPTICA Photonics), and a 100× TIRF UapoN oil objective (NA 1.49).

### Data analysis

Densitometric analysis of immunoblot data was conducted using Image Lab software (Bio-Rad), and densitometric values for protein expression were normalized to the GAPDH loading control. Statistical analysis and data plotting were performed using GraphPad Prism version 8.0 for Mac (GraphPad Software, www.graphpad.com). Figures were assembled using Pixelmator Pro for Mac (www.pixelmator.com). Gestational profile immunoblot data were subjected to a one-way analysis of variance (ANOVA) and post-hoc Tukey–Kramer multiple comparisons tests, while uterine distension immunoblot data and co-localization quantitation data were subjected to unpaired *t*-tests. Values with a *P* < 0.05 were considered significantly different.

## Results

### pS86-HSPB1 in rat myometrium

**Fig. 1:**
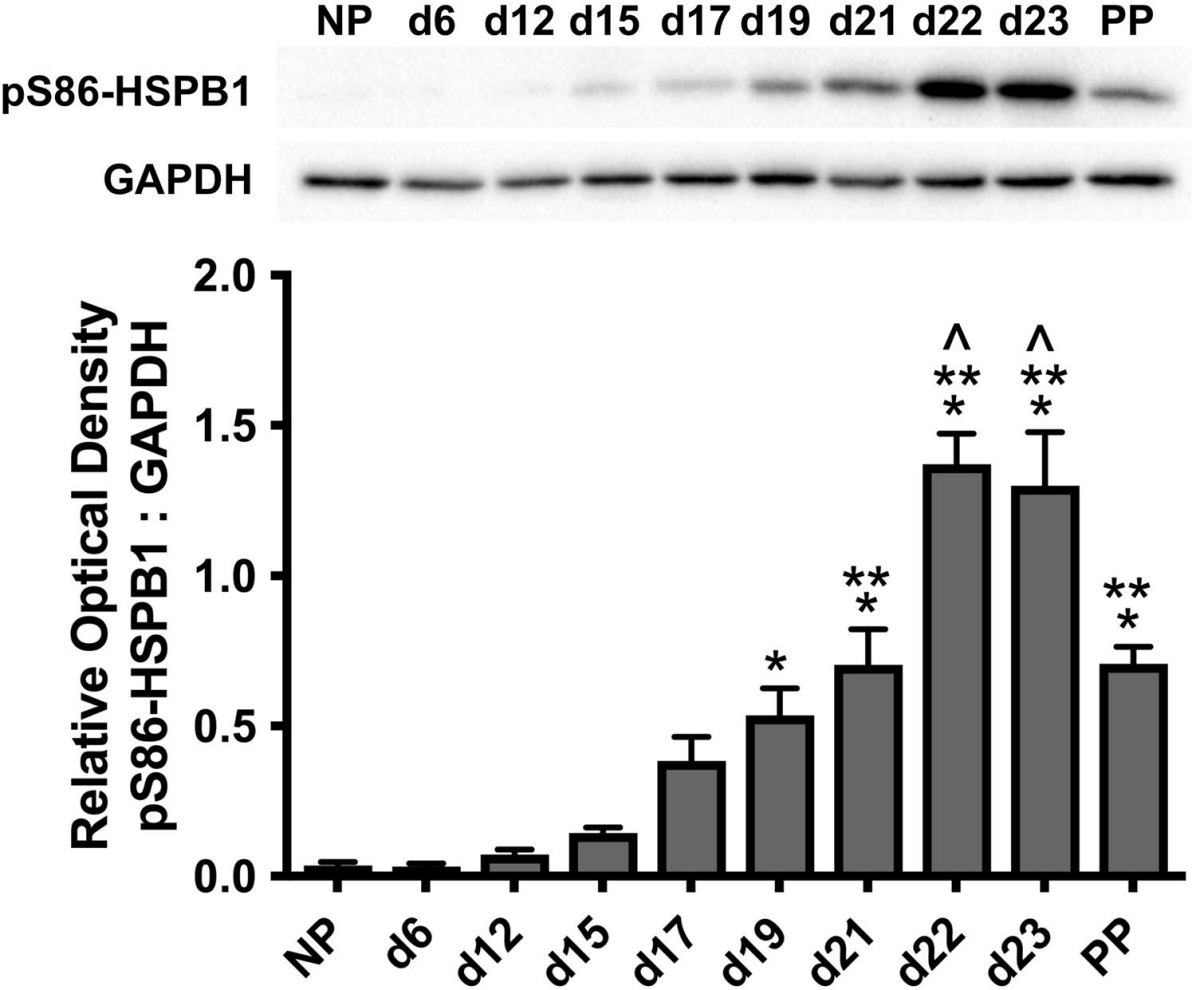
Levels of serine-86 phosphorylated HSPB1 (pS86-HSPB1) in myometrium from non-pregnant rats, during pregnancy, as well as post-partum (PP) assessed by immunoblot analysis. Representative immunoblots of pS86-HSPB1 and glyceraldehyde 3-phosphate dehydrogenase (GAPDH) are shown. Detection at day (d)19-PP was significantly elevated compared with NP-d12 (*P < 0.05) and at d21-PP compared with d15 (**P < 0.05). Detection of pS86-HSPB1 was also significantly increased at d22 and d23 compared with d17-d21 and PP (^*P* < 0.05). Mean densitometric values plotted are from four independent experiments (*n* = 4), and error bars represent the standard error of the mean (SEM)

Detection of pS86-HSPB1 in myometrium from non-pregnant rats, during pregnancy, as well as post-partum was assessed by immunoblot analysis, and pS86-HSPB1 increased markedly at late gestation and labour (Fig. 1). Specifically, detection at d19-1PP was significantly elevated compared with NP-d12 (*P < 0.05) and at d21-PP compared with d15 (**P < 0.05). Lastly, detection of pS86-HSPB1 was significantly increased at d22 and d23 compared with d17–d21 and PP (^*P* < 0.05).

**Fig. 2:**
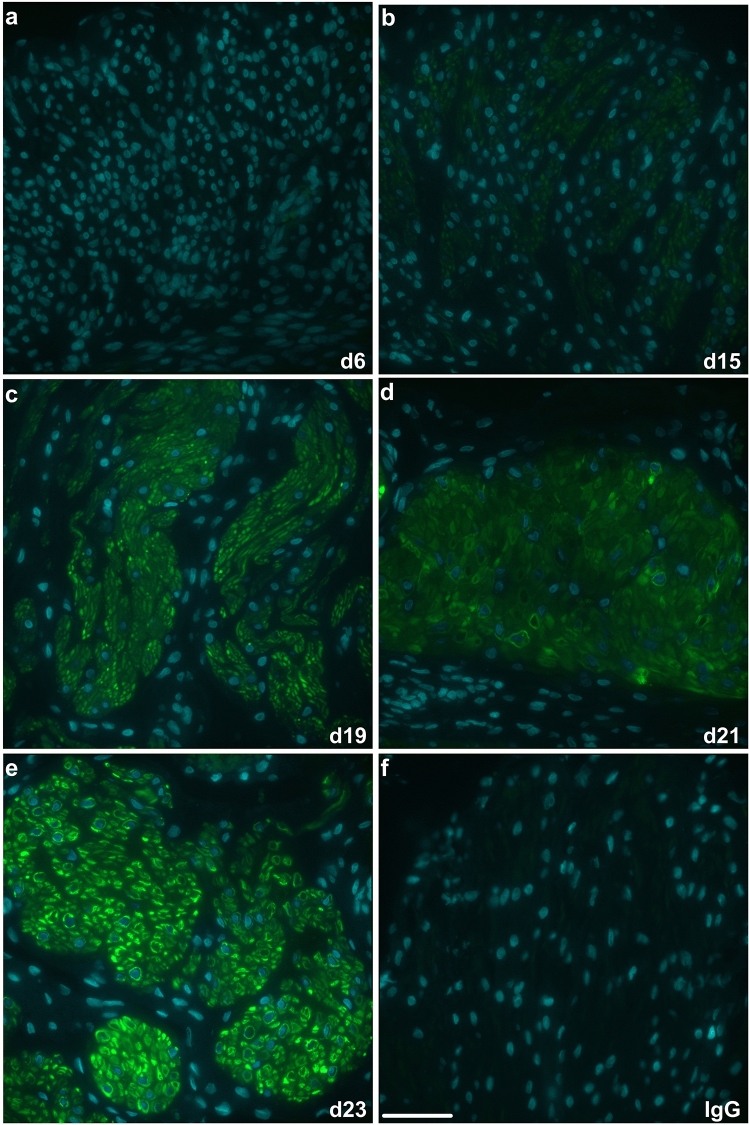
Immunofluorescence detection of serine-86 phosphorylated HSPB1 (pS86-HSPB1) in the longitudinal muscle layer of the rat myometrium during pregnancy and parturition. Representative images from day (d)6, d15, d19, d21 and d23 are shown (**a-e**). pS86-HSPB1 was mainly localized to the myocyte cytoplasm (green), with some detection associated with myocyte membranes. Detection became very prominent in late pregnancy and at d23 (labour). Nuclei were stained with DAPI (cyan). IgG, non-specific rabbit IgG control (**f**). Scale bar, 50 μm

**Fig. 3:**
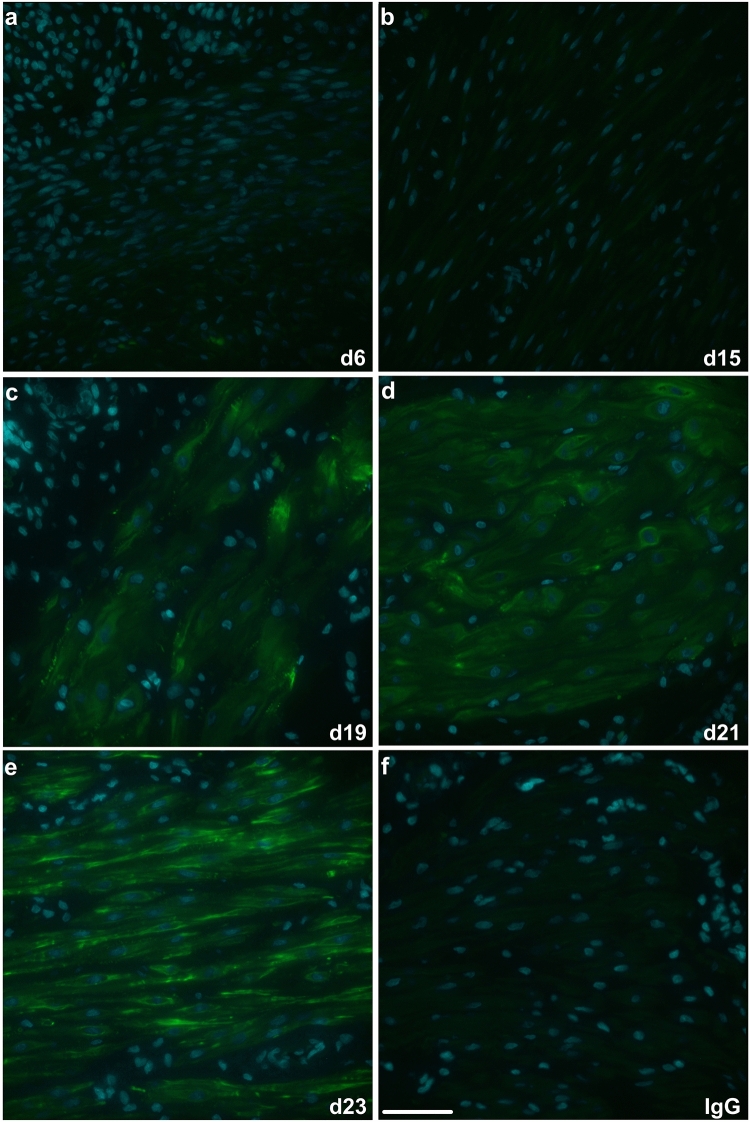
Immunofluorescence detection of serine-86 phosphorylated HSPB1 (pS86-HSPB1) in the circular muscle layer of the rat myometrium during pregnancy and parturition. Representative images from day (d)6, d15, d19, d21 and d23 are shown (**a**–**e**). pS86-HSPB1 was primarily localized to the myocyte cytoplasm (green), with some detection associated with myocyte membranes. Detection became very prominent in late pregnancy and at d23 (labour). Nuclei were stained with DAPI (cyan). IgG, non-specific rabbit IgG control (**f**). Scale bar, 50 μm

The in situ detection of pS86-HSPB1 in rat longitudinal and circular muscle layers of the myometrium was assessed by immunofluorescence analysis (Figs. 2a-e and 3a–e). The phosphorylated form of HSPB1 was virtually undetectable in rat myometrial layers until d19 (Fig. 2c, 3c) and then became prominent at d22 and d23 (Figs. 2e and 3e). In both muscle layers, pS86-HSPB1 largely exhibited cytoplasmic localization around myometrial cell nuclei (Figs. 2c-e and 3c–e).

**Fig. 4:**
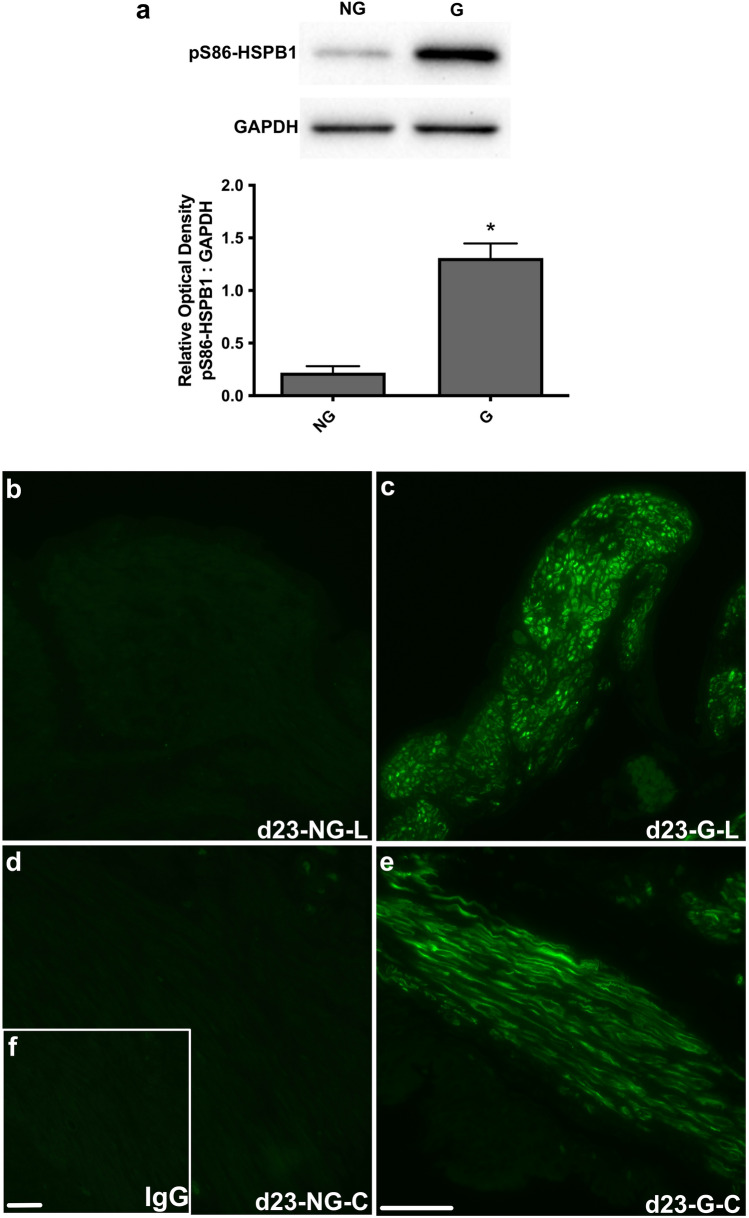
Uterine distension induces serine-86 phosphorylated HSPB1 (pS86-HSPB1) detection in rat myometrium at labour. Representative immunoblots of pS86-HSPB1 and glyceraldehyde 3-phosphate dehydrogenase (GAPDH) are provided for day (d)23 (**a**). Detection of pS86-HSPB1 on d23 in the gravid horn myometrium (G) was significantly higher (*; *P* < 0.05) compared with the non-gravid horn (NG). Densitometric values plotted are means from four independent experiments (*n* = 4), and error bars represent the standard error of the mean (SEM). Immunofluorescence analysis of pS86-HSPB1 is shown for both the longitudinal (L; **b, c**) and circular (C; **d, e**) muscle layers of the rat myometrium at d23. Detection of pS86-HSPB1 was much more prominent in the gravid horn myometrium (G) compared with the non-gravid horn (NG). IgG, non-specific rabbit IgG control (**f**). Scale bars, 50 μm

To assess the role uterine distension may have on the markedly increased detection of pS86-HSPB1 at late pregnancy and labour, immunoblot analysis was conducted on rat myometrium samples from a unilateral pregnancy model at d19 (Supplementary Fig. 1a) and d23 (Fig. 4a). Phosphorylated HSPB1 was significantly elevated in the gravid rat uterine myometrium compared with the non-gravid tissue at both timepoints (*P < 0.05). Similarly, immunofluorescence analysis demonstrated that detection of the phosphorylated HSPB1 was markedly greater in the gravid uterine myometrium at d19 (Supplementary Fig. 1b–e) and d23 (Fig. 4b–e) compared with the non-gravid in both circular and longitudinal muscle layers.

### pS82-HSPB1 in hTERT human myometrial cells

**Fig. 5:**
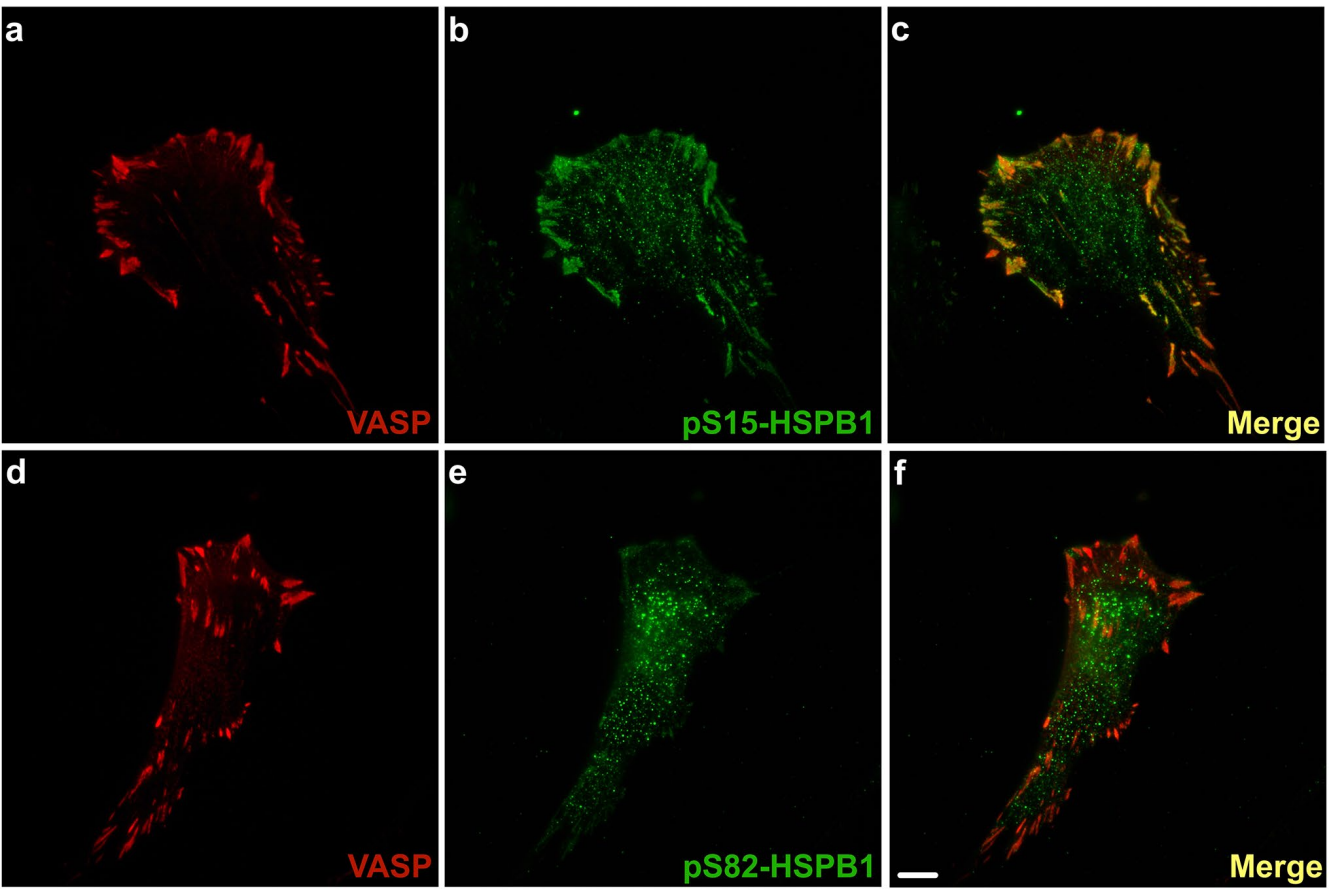
Assessment of focal adhesion localization of serine-15 and serine-82 phosphorylated forms of HSPB1 in hTERT human myometrial cells using total internal reflection microscopy. Serine-15 phosphorylated HSPB1 (pS15-HSPB1) was mainly associated with focal adhesions marked by vasodilator-stimulated phosphoprotein (VASP; **a**–**c**), while serine-82 phosphorylated HSPB1 (pS82-HSPB1) remained localized in the cytoplasm (**d**–**f**). Scale bar, 10 μm

A comparison of the subcellular localization of pS15-HSPB1 and pS82-HSPB1 (homologous to rodent pS86-HSPB1) proteins was assessed in hTERT-HM cells. Using vasodilator-stimulated phosphoprotein (VASP) as a focal adhesion marker, TIRF microscopy demonstrated that pS15-HSPB1 was prominently localized to these cell–ECM adhesions, with low levels in the cytoplasm (Fig. 5a–c), while pS82-HSPB1 was primarily located in the cell cytoplasm (Fig. 5d–f).

**Fig. 6:**
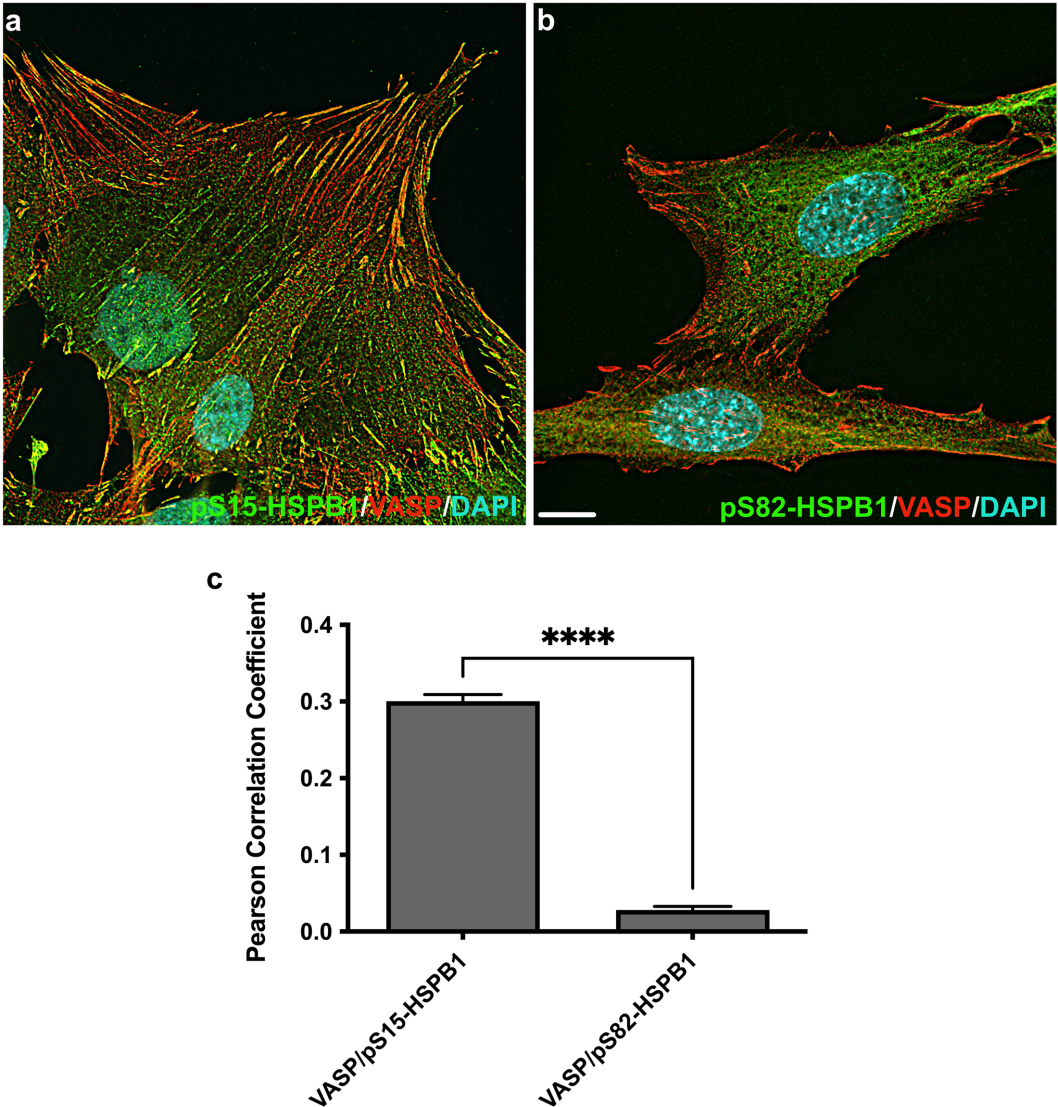
Assessment of focal adhesion localization of serine-15 and serine-82 phosphorylated forms of HSPB1 in hTERT human myometrial cells using epifluorescence microscopy and post-image acquisition deconvolution. Serine-15 phosphorylated HSPB1 (pS15-HSPB1) was mainly associated with focal adhesions marked by vasodilator-stimulated phosphoprotein (VASP; yellow; **a**), while serine-82 phosphorylated HSPB1 (pS82-HSPB1) remained localized in the cytoplasm (**b**). Nuclei were stained with DAPI (cyan). Scale bar, 10 μm. The presence of phosphorylated forms of HSPB1 with VASP was assessed using co-localization analysis and generation of Pearson correlation coefficients (**c**). VASP and pS15-HSPB1 were significantly co-localized compared to VASP and pS82-HSPB1 (****p < 0.0001)

When hTERT-HM cells were examined as a series of *z*-stacks with high magnification widefield epifluorescence microscopy followed by post-image acquisition deconvolution, pS15-HSPB1 was again chiefly localized to VASP immunostained focal adhesion structures (Fig. 6a). In contrast, pS82-HSPB1 was cytoplasmic, particularly around cell nuclei and rarely observed at focal adhesions (Fig. 6b). A more quantitative comparison of the presence of phosphorylated forms of HSPB1 with VASP-immunostained focal adhesions, using co-localization analysis and generation of Pearson correlation coefficients, confirmed the marked differences in localization of the two phosphorylated forms of HSPB1 at these sites in adhered cells (Fig. 6c).

### Comparison of pS15- and pS86-HSPB1 localization in labouring rat myometrium

**Fig. 7:**
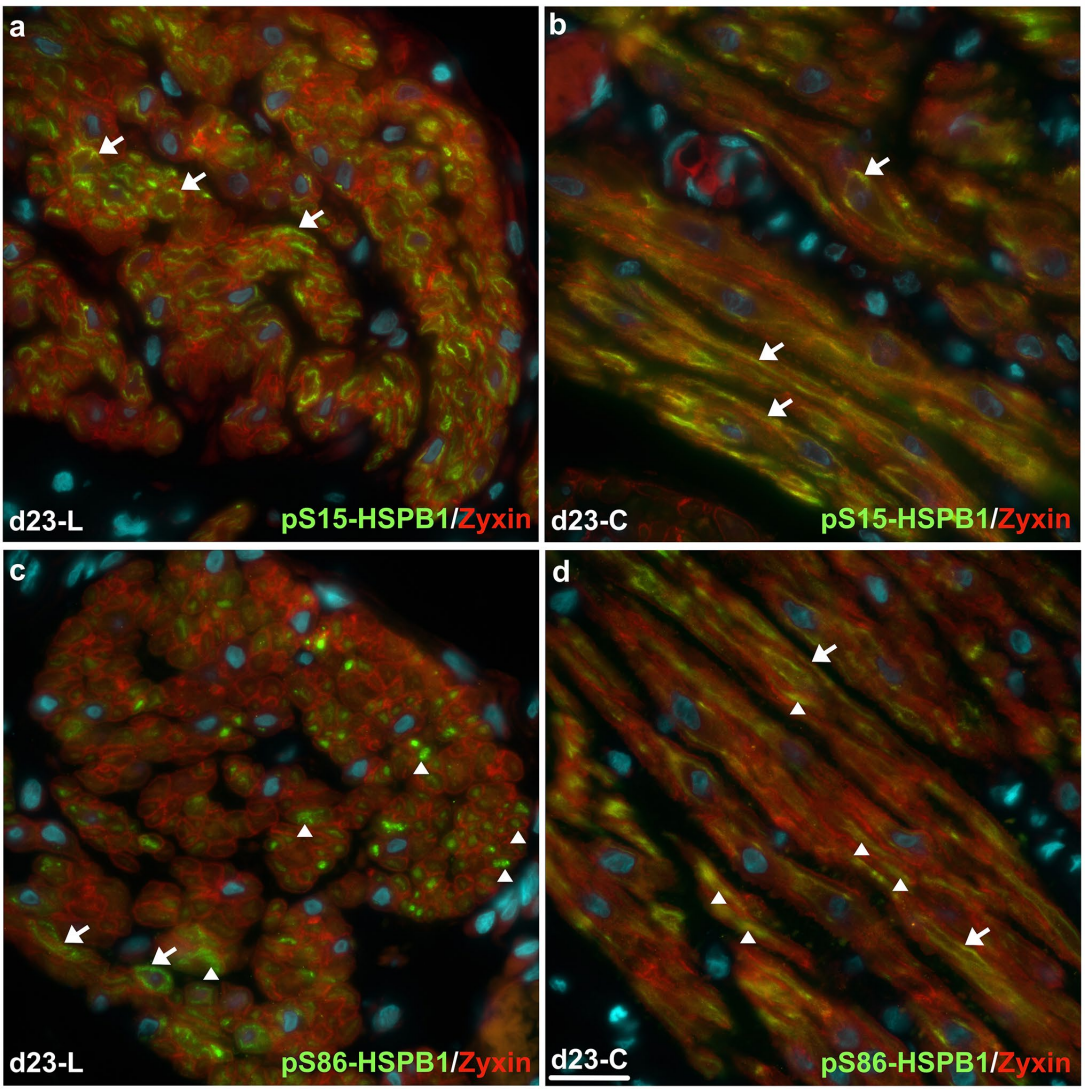
Comparison of immunofluorescence detection of serine-15 and serine-86 phosphorylated forms of HSPB1 in rat myometrium at labour. At day (d)23 of pregnancy, serine-15 phosphorylated HSPB1 (pS15-HSPB1) was mainly localized in myocyte membrane associated regions with zyxin (arrows) in both longitudinal (**a**) and circular layers (**b**). In contrast, serine-86 phosphorylated HSPB1 (pS86-HSPB1) was predominantly localized to the myocyte cytoplasm (arrowheads) in both layers (**c** and **d**), with some detection associated with myocyte membranes (arrows). L, longitudinal muscle layer; C, circular muscle layer. Nuclei were stained with DAPI (cyan). Scale bar, 25 μm

The localization of the two phosphorylated forms of HSPB1 was also compared in labouring rat myometrium. Focal adhesions were marked by zyxin detection as zyxin and VASP co-localize to focal adhesions in uterine smooth muscle cells (Supplementary Fig. 2a–d). Detection of pS15-HSPB1 was primarily membrane-associated with zyxin in both longitudinal and circular muscle layers (Fig. 7a,b), while pS86-HSPB1 was localized in the cytoplasm of myocytes in the longitudinal and circular muscle layers (Fig. 7c,d). However, pS86-HSPB1 could also be detected in some membrane-associated regions with zyxin.

## Discussion

Phosphorylation of some HSPB members on serine residues is critical for regulation of their structure and function and is known to be one of the earliest events induced by stress (Landry et al. 1992; Mymrikov et al. 2011; Jovacevski et al. 2015). Within the NH_2_-terminal region of HSPB1, Ser-15 is within the conserved hydrophobic WDPF motif, while Ser-78 and Ser-82 (humans; only Ser-86 in rats) are within the connecting peptide to the C-terminal domain (Lambert et al. 1999). The use of mutations in human HSPB1 mimicking phosphorylation demonstrated that any single phosphorylated form of HSPB1 increased the detection of HSPB1 dimers and enhanced chaperone activity compared with oligomers, both of which significantly increased upon double or triple Ser phosphorylation (Jovacevski et al. 2015, 2017). However, examination of phosphomimicking forms of Chinese hamster HSPB1, lacking a homologous Ser-78 residue like mice and rats, demonstrated that only phosphorylation of Ser-90 (Ser-86 in rats; Ser-82 in humans) decreased oligomer size and enhanced chaperone activity, highlighting the importance of this specific phosphorylation site (Lambert et al. 1999).

In our study, high levels of pS86-HSPB1 were detected in late pregnant and labouring rat myometrium, correlating with previous work showing significant levels of pS15-HSPB1 between d19 and d23 in the same animal model (White et al. 2005). However, pS86-HSPB1 levels remained significantly elevated PP unlike pS15-HSPB1. Prominent detection of pS86-HSPB1 in situ at d22-d23 also correlated with previous detection of pS15-HSPB1 in uterine muscle layers. Lastly, rat uterine stretch significantly increased levels of pS86-HSPB1 similarly to pS15-HSPB1 (White and MacPhee 2011).

Despite similarities in timing of detection over pregnancy, there were marked differences in spatial localization of the two phosphorylated forms of HSPB1 in the myometrium. In the present study, pS86-HSPB1 localization was largely cytoplasmic around myometrial cell nuclei, while pS15-HSPB1 was more commonly associated with myocyte membranes during late pregnancy and labour as previously shown by White et al. (2005). Nevertheless, we cannot rule out that at least some of the HSPB1 detected in these studies was phosphorylated on both serine residues. Thus, these data highlight that elevated detection of serine phosphorylated HSPB1 is associated with late pregnancy and labour in the rat myometrium and that uterine distension is a major stress-inducing signal for phosphorylation of this small stress protein.

Our data differ slightly from previous work by MacIntyre and colleagues (2008) in human myometrium that showed pS15-HSPB1 levels were similarly induced in women in established labour (at least 2 h) compared with a non-labouring (caesarean section) group. However, pS82-HSPB1 levels were significantly reduced in the labouring compared with the non-labouring group. The latter data may reflect a species-specific difference as pS78-HSPB1 levels were maintained at a highly detectable level in both labouring and non-labouring groups. Thus, pS78-HSPB1 in human myometrium may aid HSPB1 chaperone function and compensate for any decreased levels of pS82-HSPB1, while pS86-HSPB1 could play that exclusive role in rat myometrium.

The apparent differences in location of pS15-HSPB1 and pS86-HSPB1 observed in the rat uterine myocytes in situ imply that specific duties exist for these phosphorylated forms within the myometrium during late pregnancy and labour. To address these differences, we examined the localization of these two HSPB1 forms more meticulously in an immortalized human myometrial cell line using TIRF microscopy that focuses on a depth of approximately 100 nm from the plasma membrane and widefield epifluorescence imaging of myocyte *z*-stacks coupled with deconvolution for examination of greater optical depths. These experiments demonstrated that pS15-HSPB1 was prominently localized to focal adhesions, marked by VASP staining, while pS82-HSPB1 was primarily located in the cell cytoplasm.

**Fig. 8:**
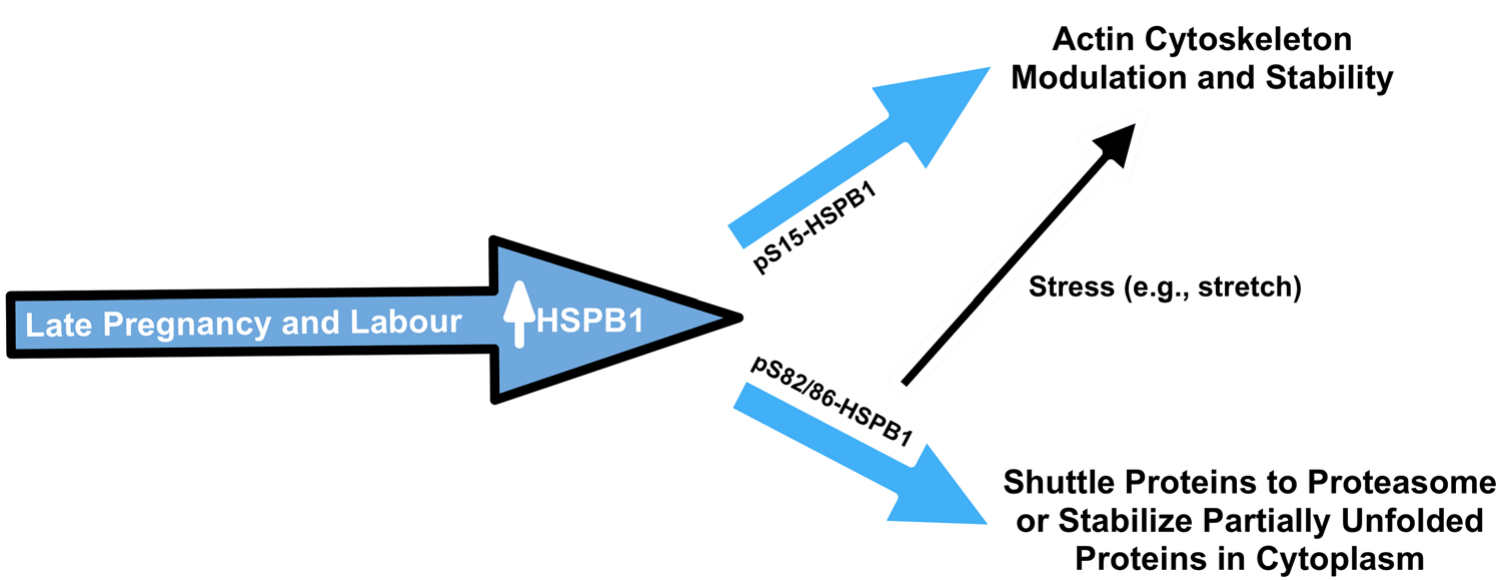
Proposed model for HSPB1 function in the myometrium during late pregnancy and labour. The significantly increased levels of serine-15 phosphorylated HSPB1 (pS15-HSPB1) in the myometrium during late pregnancy and labour could be involved in regulation of actin filament client modulation and stability while serine-82/86 phosphorylated HSPB1 (pS82/86-HSPB1) could shuttle proteins to the proteasome or stabilize partially unfolded cytoplasmic proteins needed for late gestation and labour. When required, as during a specific stress, pS82/86-HSPB1 may still assist with modulation or stability of clients such as actin at cell–extracellular matrix adhesion sites

Focal adhesions are stratified layers of many distinct proteins that in concert transmit mechanical forces from integrin receptors to actin filaments. VASP and zyxin are proteins that localize to these focal adhesions at stress fibres, forming comet tails in cultured cells (Guo and Wang 2007), and are important in actin polymerization, repair and stabilization (Beckerle 1986; Smith et al. 2010; Faix and Rottner 2022). In fact, VASP recruitment to focal adhesions requires zyxin (Hoffman et al. 2006). The repair and stabilization of actin filaments at focal adhesions during late pregnancy and labour would be important for myocytes to be able to modulate their cytoskeleton in response to distension and generate contractile forces as a tissue. Overexpression of HSPB1 in rodent fibroblasts was demonstrated to increase stress fibre stability (Lavoie et al. 1993a). Additionally, it was reported that overexpression of human HSPB1 in Chinese hamster cell lines led to increased levels of F-actin, while a non-phosphorylatable HSPB1 mutant decreased levels of F-actin at the cell cortex (Lavoie et al. 1993b); however, the study never examined the specific HSPB1 phosphorylation sites involved. In rat pheochromocytoma PC12 cells, stress (heat shock) significantly increased the interaction of pS15-HSPB1 with F-actin (Clarke and Mearow 2013). Thus, the localization of pS15-HSPB1 to VASP-immunostained focal adhesions in hTERT-HM cells as well as in membrane-associated regions of myometrial cells in situ in our study suggests a role for this form of HSPB1, likely with VASP, in regulation of actin filament modulation and stability in myometrial cells at cell–ECM sites (Fig. 8).

When HeLa cells were examined for the distribution of phosphorylated forms of HSPB1 using size exclusion chromatography, the presence of pS15-HSPB1 and pS82-HSPB1 within small and large fractions differed significantly, suggesting distinct functions for these phosphorylated forms (Arrigo 2017). Our detection of pS82-HSPB1 in the cytoplasm of hTERT-HM cells and in rat myometrium in situ, in contrast to pS15-HSPB1, also supports this supposition. The presence of pS82-HSPB1 in the cytoplasm has also been reported in human neutrophils and HeLa cells, but pS15-HSPB1 localization was not examined (During et al. 2007).

At this time, we can only speculate on the role of cytoplasmic pS82/86-HSPB1 in myometrial cells. Parcellier et al. (2003) reported that HSPB1 was a ubiquitin-binding cytoplasmic protein involved in proteasomal degradation of Inhibitor of kappa B which can lead to activation of the inflammatory mediator Nuclear Factor kappa B. Thus, the cytoplasmic pS82/86-HSPB1 we observed in rat and human myometrial cells could be involved in shuttling proteins to the proteasome (Fig. 8) to facilitate parturition. Nonetheless, the specific organelles, cytoplasmic components and/or client proteins associated with pS82/86-HSPB1 in myometrial cells need to be determined.

Recently, it was reported that pS86-HSPB1 in mouse fibroblasts was induced to localize to stress fibres at comet tails, marked by zyxin localization, after uniaxial cyclic stretch in vitro while unstretched cells displayed cytoplasmic pS86-HSPB1 (Hoffman et al. 2017). However, the localization of pS15-HSPB1 was not specifically examined in that study. Our own examination of pS15- and pS86-HSPB1 in situ in labouring rat myometrium demonstrated that pS86-HSPB1 was localized mainly to the cytoplasm but there was some association with myocyte membranes. Thus, under stress such as uterine distension and/or the labour process itself, some pS86-HSPB1 could be recruited to client proteins, such as the actin cytoskeleton or other focal adhesion proteins, for specific needs when required (Fig. 8).

## Supplementary Information

Below is the link to the electronic supplementary material.

Supplementary Fig. 1 Uterine distension induces serine-86 phosphorylated (pS86-HSPB1) HSPB1 detection in rat myometrium at day 19 of pregnancy. Representative immunoblots of pS86-HSPB1 and glyceraldehyde 3-phosphate dehydrogenase (GAPDH) are provided for day (d)19 (a). Detection of pS86-HSPB1 on d19 in the gravid horn myometrium (G) was significantly higher (*; *P* < 0.05) compared to the non-gravid horn (NG). Densitometric values plotted are means from four independent experiments (*n* = 4) and error bars represent the standard error of the mean (SEM). Immunofluorescence analysis of pS86-HSPB1 is shown for both the longitudinal (L; b, c) and circular (C; d, e) muscle layers of the rat myometrium at d19. Detection of pS86-HSPB1 was much more prominent in the gravid horn myometrium (G) compared to the non-gravid horn (NG). IgG, non-specific rabbit IgG control (f). Scale bars = 50 μm (JPG 2458 KB)

Supplementary Fig. 2 Co-localization of vasodilator-stimulated phosphoprotein (VASP) and zyxin in focal adhesions and comet tails of hTERT human myometrial cells. Following widefield epifluorescence multiple imaging of z-stacks (5 × 0.25 μm thick) for VASP (a), zyxin phosphorylated on Ser-142/143 (Cell Signaling Technology, #8467, 1:100 dilution) which localizes at focal adhesions and comet tails (b) and actin (Phalloidin-AF647; ThermoFisher, #A22287; 1:20 dilution) (c), images underwent deconvolution with 5 repetitions per slice of constrained iterative restoration using the advanced maximum likelihood estimation algorithm. VASP and zyxin expression were highly co-localized. Nuclei were stained with DAPI (magenta). Scale bar = 10 μm (JPG 4335 KB)

## Data Availability

Data can be made available upon reasonable request.

## References

[CR1] Acunzo J, Katsogiannou M, Rocchi P (2012). Small heat shock proteins HSP27 (HSPB1), alphaB-crystallin (HSPB5) and HSP22 (HSPB8) as regulators of cell death. Int J Biochem Cell Biol.

[CR2] Arrigo A-P (2017). Mammalian HspB1 (Hsp27) is a molecular sensor linked to the physiology and environment of the cell. Cell Stress Chap.

[CR3] Bhatti M, Dinn S, Miskiewicz EI, MacPhee DJ (2019). Expression of heat shock factor 1, heat shock protein 90 and associated signaling proteins in pregnant rat myometrium: implications for myometrial proliferation. Reprod Biol.

[CR4] Beckerle MC (1986). Identification of a new protein localized at sites of cell–substrate adhesion. J Cell Biol.

[CR5] Benndorf R, Hayess K, Ryazantsev S, Wieske M, Behlke J, Lutsch G (1994). Phosphorylation and supramolecular organization of murine small heat shock protein HSP25 abolish its actin polymerization inhibiting activity. J Biol Chem.

[CR6] Bonney EA (2013). Demystifying animal models of adverse pregnancy outcomes: touching bench and bedside. Am J Reprod Immunol.

[CR7] Clarke JP, Mearow KM (2013). Cell stress promotes the association of phosphorylated HspB1 with F-actin. PLoS ONE.

[CR8] Collier MP, Benesch JLP (2020). Small heat-shock proteins and their role in mechanical stress. Cell Stress Chap.

[CR9] Condon J, Yin S, Mayhew B, Word RA, Wright WE, Shay JW, Rainey WE (2002). Telomerase immortalization of human myometrial cells. Biol Reprod.

[CR10] During RL, Gibson BG, Li W, Bishai EA, Sidhu GS, Landry J, Southwick FS (2007). Anthrax lethal toxin paralyzes actin-based motility by blocking hsp27 phosphorylation. EMBO J.

[CR11] Faix J, Rottner K (2022). Ena/VASP proteins in cell edge protrusion, migration and adhesion. J Cell Sci.

[CR12] Fontaine JM, Sun X, Benndorf R, Welsh MJ (2005). Interactions of HSP22 (HSPB8) with HSP20, alphaB-crystallin, and HSPB3. Biochem Biophys Res Commun.

[CR13] Gaestel M, Schroder W, Benndorf R, Lippmann C, Buchner K, Hucho F, Erdmann VA, Bielka H (1991). Identification of the phosphorylation sites of the murine small heat shock protein Hsp25. J Biol Chem.

[CR14] Guay J, Lambert H, Gingras-Breton G, Lavoie JN, Huot J, Landry J (1997). Regulation of actin filament dynamics by p38 map kinase-mediated phosphorylation of heat shock protein 27. J Cell Sci.

[CR15] Guo WH, Wang YL (2007). Retrograde fluxes of focal adhesion proteins in response to cell migration and mechanical signals. Mol Biol Cell.

[CR16] Haslbeck M, Weinkauf S, Buchner J, Tanguay RM, Hightower LE (2015). Regulation of the chaperone function of small hsps. The big book of small heat shock proteins.

[CR17] Hoffman LM, Jensen CC, Kloeker S, Wang CL, Yoshigi M, Beckerle MC (2006). Genetic ablation of zyxin causes Mena/VASP mislocalization, increased motility, and deficits in actin remodeling. J Cell Biol.

[CR18] Hoffman L, Jensen CC, Yoshigi M, Beckerle M (2017). Mechanical signals activate p38 MAPK pathway-dependent reinforcement of actin via mechanosensitive HspB1. Mol Biol Cell.

[CR19] Ibitayo AI, Sladick J, Tuteja S, Louis-Jacques O, Yamada H, Groblewski G, Welsh M, Bitar KN (1999). Hsp27 in signal transduction and association with contractile proteins in smooth muscle cells. Am J Physiol.

[CR20] Jovcevski B, Kelly MA, Rote AP, Berg T, Gastall HY, Benesch JLP, Aquilina JA, Ecroyd H (2015). Phosphomimics destabilize Hsp27 oligomeric assemblies and enhance chaperone activity. Chem Biol.

[CR21] Jovcevski B, Kelly MA, Aquilina JA, Benesch JLP, Ecroyd H (2017). Evaluating the effect of phosphorylation on the structure and dynamics of Hsp27 dimers by means of ion mobility mass spectrometry. Anal Chem.

[CR22] Kampinga HH, Garrido C (2012). HSPBs: small proteins with big implications in human disease. Int J Biochem Cell Biol.

[CR23] Kostenko S, Moens U (2009). Heat shock protein 27 phosphorylation: kinases, phosphatases, functions and pathology. Cell Mol Life Sci.

[CR24] Lambert H, Charette SJ, Bernier AF, Guimond A, Landry J (1999). HSP27 multimerization mediated by phosphorylation-sensitive intermolecular interactions at the amino terminus. J Biol Chem.

[CR25] Landry J, Lambert H, Zhou M, Lavoie JN, Hickey E, Weber LA, Anderson CW (1992). Human HSP27 is phosphorylated at serines 78 and 82 by heat shock and mitogen-activated protein kinases that recognize the same amino acid motif as S6 kinase II. J Biol Chem.

[CR26] Lavoie JN, Gingras-Breton G, Tanguay RM, Landry J (1993). Induction of Chinese hamster Hsp27 gene expression in mouse cells confers resistance to heat shock. Hsp27 stabilization of the microfilament organization. J Biol Chem.

[CR27] Lavoie JN, Hickey E, Weber LA, Landry J (1993). Modulation of actin microfilament dynamics and fluid phase pinocytosis by phosphorylation of heat shock protein 27. J Biol Chem.

[CR28] MacIntyre DA, Tyson EK, Read M, Smith R, Yeo G, Kwek K, Chan EC (2008). Contraction in human myometrium is associated with change in small heat shock proteins. Endocrinology.

[CR29] MacPhee DJ, Miskiewicz EI (2017). The potential functions of small heat shock proteins in the uterine musculature during pregnancy. Adv Anat Embryol Cell Biol.

[CR30] Menon R, Bonney EA, Condon J, Mesiano S, Taylor RN (2016). Novel concepts on pregnancy clocks and alarms: redundancy and synergy in human parturition. Hum Reprod Update.

[CR31] Mitchell BF, Taggart MJ (2009). Are animal models relevant to key aspects of human parturition?. Am J Physiol.

[CR32] Mymrikov EV, Seit-Nebi AS, Gusev NB (2011). Large potentials of small heat shock proteins. Physiol Rev.

[CR33] Nicoletti JG, White BG, Miskiewicz EI, MacPhee DJ (2016). Induction of expression and phosphorylation of heat shock protein B5 (CRYAB) in rat myometrium during pregnancy and labour. Reproduction.

[CR34] Oldenhof AD, Shynlova OP, Liu M, Langille BL, Lye SJ (2002). Mitogen-activated protein kinases mediate stretch-induced c-fos mRNA expression in myometrial smooth muscle cells. Am J Physiol Cell Physiol.

[CR35] Ou C-W, Orsino A, Lye SJ (1997). Expression of connexin-43 and connexin-26 in the rat myometrium during pregnancy and labor is differentially regulated by mechanical and hormonal signals. Endocrinology.

[CR36] Parcellier A, Schmitt E, Gurbuxani S, Seigneurin-Berny D, Pance A, Chantome A, Plenchette S, Khochbin S, Solary E, Garrido C (2003). HSP27 is a ubiquitin-binding protein involved in I-kBα proteasomal degradation. Mol Cell Biol.

[CR37] Shynlova O, Lee Y-H, Srikhajon K, Lye SJ (2013). Physiologic uterine inflammation and labor onset: integration of endocrine and mechanical signals. Reprod Sci.

[CR38] Shynlova O, Nadeem L, Zhang J, Dunk C, Lye SJ (2020). Myometrial activation: novel concepts underlying labor. Placenta.

[CR39] Smith MA, Blankman E, Gardel ML, Luettjohann L, Waterman CM, Beckerle MC (2010). A zyxin-mediated mechanism for actin stress fiber maintenance and repair. Dev Cell.

[CR40] van Noort JM, Bsibsi M, Nacken P, Gerritsen WH, Amor S (2012). The link between small heat shock proteins and the immune system. Int J Biochem Cell Biol.

[CR41] Wettstein G, Bellaye PS, Micheau O, Bonniaud P (2012). Small heat shock proteins and the cytoskeleton: an essential interplay for cell integrity?. Int J Biochem Cell Biol.

[CR42] White BG, Williams SJ, Highmore K, MacPhee DJ (2005). Small heat shock protein 27 (Hsp27) expression is highly induced in rat myometrium during late pregnancy and labour. Reproduction.

[CR43] White BG, MacPhee DJ (2011). Distension of the uterus induces HspB1 expression in rat uterine smooth muscle. Am J Physiol.

[CR44] Zantema A, Verlaan-De Vries M, Maasdam D, Bol S, van der Eb A (1992). Heat shock protein 27 and alpha B-crystallin can form a complex, which dissociates by heat shock. J Biol Chem.

