# Supplementary figures and images for: Phosphoserine-86-HSPB1 (pS86-HSPB1) is cytoplasmic and highly induced in rat myometrium at labour
Source: Histochem Cell Biol. 2022 Oct 19;159(2):149–62. doi: 10.1007/s00418-022-02158-1 (PMC9922239; doi:10.1007/s00418-022-02158-1)

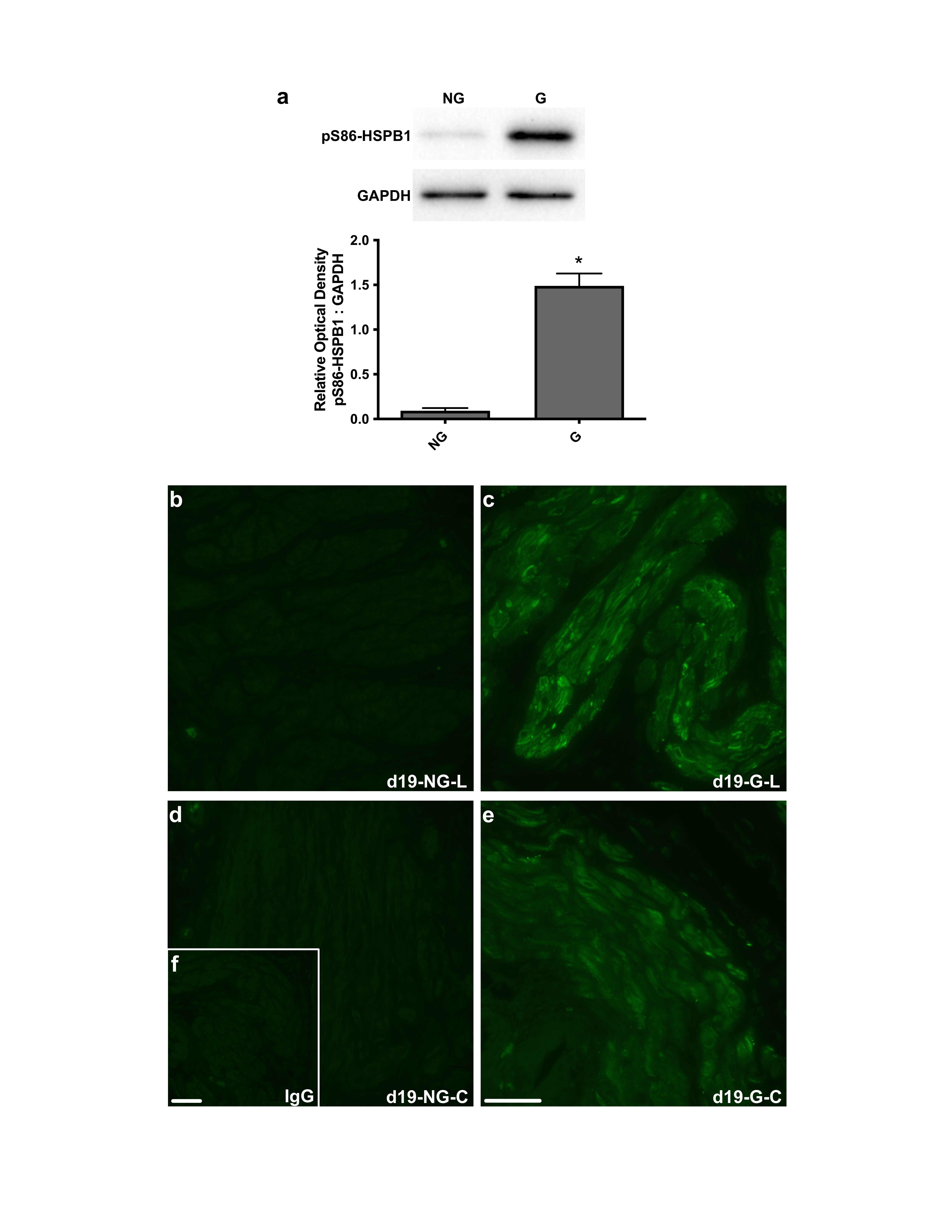

Supplement: Supplementary file 1 — Supplementary Fig. 1 Uterine distension induces serine-86 phosphorylated (pS86-HSPB1) HSPB1 detection in rat myometrium at day 19 of pregnancy. Representative immunoblots of pS86-HSPB1 and glyceraldehyde 3-phosphate dehydrogenase (GAPDH) are provided for day (d)19 (a). Detection of pS86-HSPB1 on d19 in the gravid horn myometrium (G) was significantly higher (*; P < 0.05) compared to the non-gravid horn (NG). Densitometric values plotted are means from four independent experiments (n = 4) and error bars represent the standard error of the mean (SEM). Immunofluorescence analysis of pS86-HSPB1 is shown for both the longitudinal (L; b, c) and circular (C; d, e) muscle layers of the rat myometrium at d19. Detection of pS86-HSPB1 was much more prominent in the gravid horn myometrium (G) compared to the non-gravid horn (NG). IgG, non-specific rabbit IgG control (f). Scale bars = 50 μm (JPG 2458 KB) [file 418_2022_2158_MOESM1_ESM.jpg]

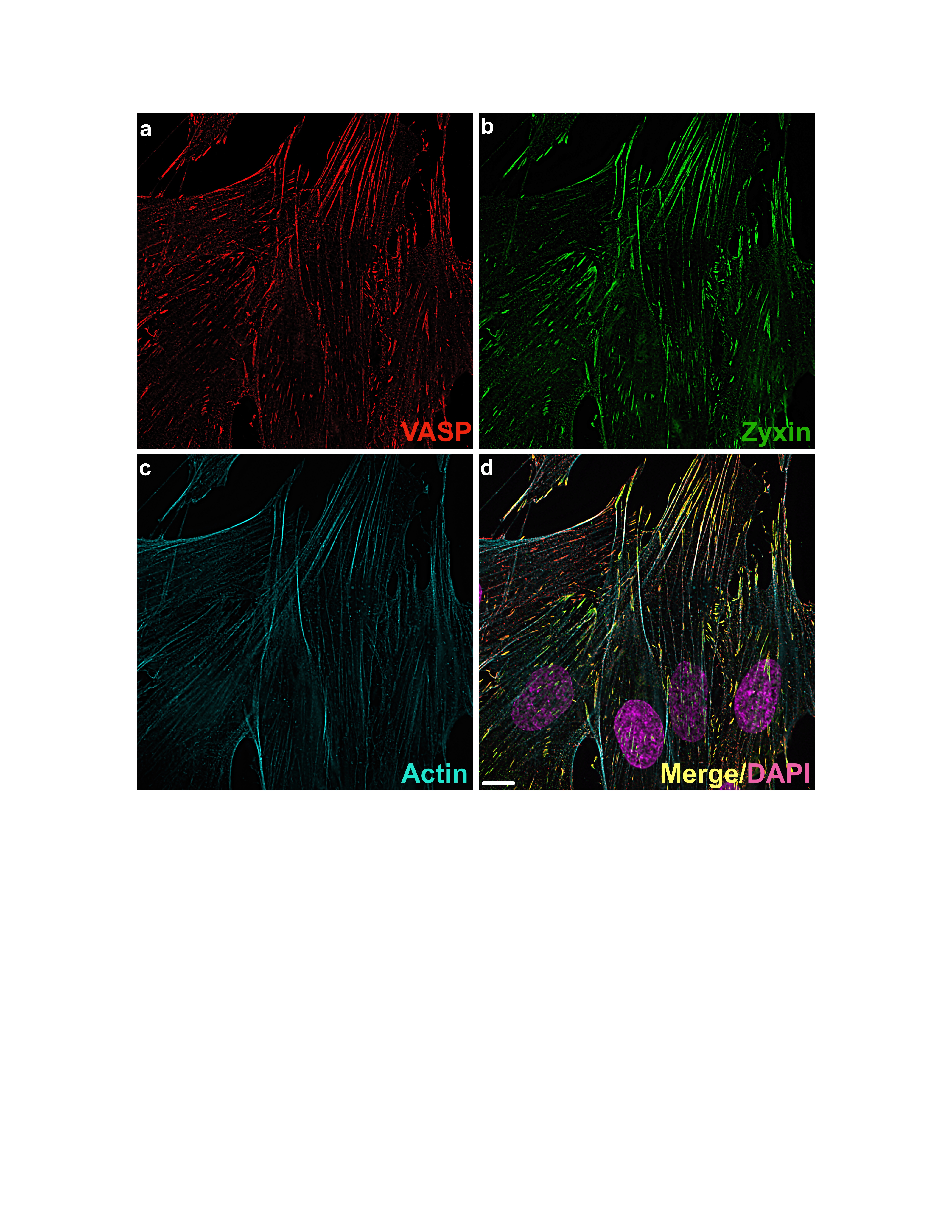

Supplement: Supplementary file 2 — Supplementary Fig. 2 Co-localization of vasodilator-stimulated phosphoprotein (VASP) and zyxin in focal adhesions and comet tails of hTERT human myometrial cells. Following widefield epifluorescence multiple imaging of z-stacks (5 × 0.25 μm thick) for VASP (a), zyxin phosphorylated on Ser-142/143 (Cell Signaling Technology, #8467, 1:100 dilution) which localizes at focal adhesions and comet tails (b) and actin (Phalloidin-AF647; ThermoFisher, #A22287; 1:20 dilution) (c), images underwent deconvolution with 5 repetitions per slice of constrained iterative restoration using the advanced maximum likelihood estimation algorithm. VASP and zyxin expression were highly co-localized. Nuclei were stained with DAPI (magenta). Scale bar = 10 μm (JPG 4335 KB) [file 418_2022_2158_MOESM2_ESM.jpg]
